# Unique Case Report of Pineal Gland Metastasis From Bladder Carcinoma

**DOI:** 10.1097/MD.0000000000003622

**Published:** 2016-05-06

**Authors:** Jun Li, Ping Wang, Bin Wang

**Affiliations:** From the Department of Radiology, Affiliated Hospital of Binzhou Medical University, Binzhou, Shandong, People's Republic of China.

## Abstract

Pineal metastasis is uncommon and most metastatic pineal lesions are asymptomatic. To our knowledge the herein reported case is the first in which the pineal gland was confirmed as the metastatic site of a bladder carcinoma.

The patient reported in this case is a 59-year-old man who suffered from headache and delirium for 4 days after surgical treatment for removal of a bladder carcinoma 1 year ago. Magnetic resonance imaging (MRI) revealed a solid tumor involving the pineal gland with significant enhancement.

The patient underwent surgical treatment for removal of the neoplastic lesion in the pineal gland. Histopathological examination confirmed invasion of the pineal gland by metastatic urothelial carcinoma.

This case highlighted that the presence of pineal lesions in patient with known malignancy should raise suspicion of metastatic involvement.

## INTRODUCTION

The pineal region is a site of predilection for primary tumors of the pineal region and germ cell tumors. Pineal region metastases are very rare, occurring in 0.4% to 3.8% of patients with solid tumors as seen on autopsy reports and in 5% of patients referred to surgical management of pineal tumors.^1,2^

Metastasis to the pineal gland is most frequently found in lung cancer.^2–4^ Previous cases also reported pineal region metastases caused by other tumors such as esophagus, pancreas, kidney, stomach, colon, melanoma, and myeloma.^1,5–7^ Here we present, to the best of our knowledge, the first case of a bladder carcinoma metastatic to the pineal gland.

## CASE REPORT

**FIGURE 1:**
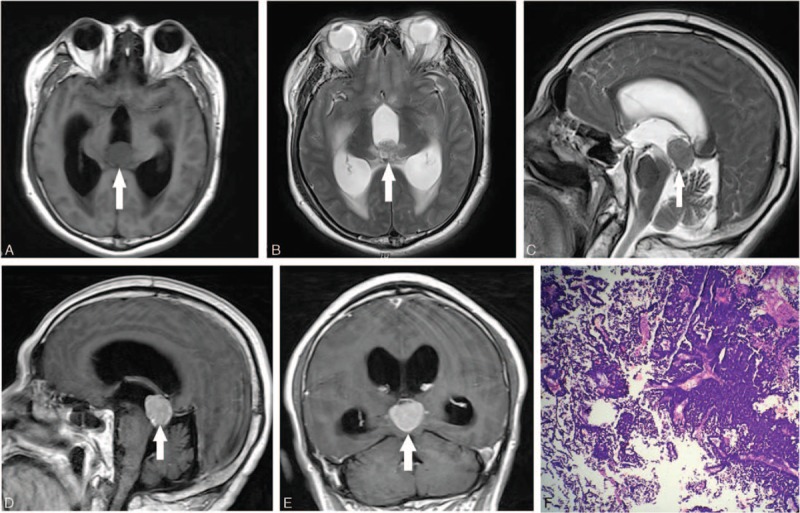
T1-weighted axial (A), T2-weighted axial (B), and T2-weighted sagittal (C) images showing the pineal region tumor (arrow). Contrast-enhanced T1-weighted images (D, E) showing the tumor (arrow) with significant enhancement. Pathological image (F) showing metastatic urothelial carcinoma with tumor cells arranged in nests.

The patient reported in this case is a 59-year-old man who suffered from headache and delirium for 4 days after surgical treatment for removal of a bladder carcinoma 1 year ago. This study was approved by Ethic Committee of Binzhou Medical University and the patient provided the written informed consent to participate in this study. This consent procedure was approved by Ethics Committee of Binzhou Medical University. Brain magnetic resonance imaging (MRI) of the patient revealed a solid tumor of 2.5 cm diameter, involving the pineal gland and abutting in the third cerebral ventricle, resulting in enlargement of the 2 lateral ones. T1-weighted axial (Figure 1A), T2-weighted axial (Figure 1B), and T2-weighted sagittal (Figure 1C) images revealed the tumor with similar signal intensity to grey matter. Contrast-enhanced T1-weighted axial (Figure 1D) and coronal (Figure 1E) images revealed significant enhancement of the tumor. Biochemical analysis of the cerebrospinal fluid revealed an increase in protein (168 mg/dL; normal: 15–45 mg/dL) level.

The patient underwent surgical treatment for removal of the neoplastic lesion in the pineal gland. The histological examination of the removed neoplastic fragments showed invasion of the pineal gland by a bladder carcinoma (Figure 1F) with immunohistochemistry results: GFAP (–), Ki-67 (+), S-100 (–), CK7 (+), CK20 (–), AFP (+), Syn (+), ADM5.2 (+), LCA (–), CD56 (–), P53 (+), and P63 (–).

After surgical resection, the hydrocephalus was not resolved and procedure of ventricular shunt was applied. However, the patient still presented delirium and was finally discharged from hospital ∼1 month postoperatively.

## DISCUSSION

Pineal metastases are generally considered uncommon and incidental events that occur late in the course of widely metastatic systemic cancer.^1^ The most commonly reported primary tumors are lung carcinomas.^2–4^ The case presented here represents, as far as we are aware, the first report of a pineal gland metastasis from a bladder carcinoma.

In the majority of cases, metastasis to the pineal gland is asymptomatic.^2^ However, this patient presented with obvious headache and delirium. Some patients can also experience other neurological symptoms such as Parinaud syndrome.^1,8^

In this study, the metastatic pineal tumor was significantly enhanced. The suggested explanation is the absence of the blood–brain barrier in the pineal gland. Metastatic disease is thought to be related to hematogenous spread because of the absence of a blood–brain barrier in the pineal gland.^2^

The pineal tumors include a wide variety of tumors, ranging from benign to highly malignant.^9^ MRI examination has important value in the differential diagnosis. Pineal cyst often presents a uniform signal similar to cerebrospinal fluid.^10^ Pineal germinoma and primary pineal tumor often show isointensity on T1-weighted and T2-weighted images. Germinoma is sensitive to radiotherapy.^11^ Pineoblastoma is highly malignant and tends to involve adjacent brain structures. Teratoma is characterized by heterogeneous signals for containing a variety of components such as calcification and fat. Papillary tumor of the pineal region often shows high-intensity signal on T1-weighted sequences.^12^

In conclusion, metastatic disease, albeit uncommon, should be considered in the differential diagnosis of pineal tumors. The presence of pineal lesions in patients with known malignancy should raise suspicion of metastatic involvement.
